# Determination of Nicotine in Tobacco by Chemometric Optimization and Cation-Selective Exhaustive Injection in Combination with Sweeping-Micellar Electrokinetic Chromatography

**DOI:** 10.1155/2015/869719

**Published:** 2015-05-26

**Authors:** Yi-Hui Lin, Chia-Hsien Feng, Shih-Wei Wang, Po-Yun Ko, Ming-Hsun Lee, Yen-Ling Chen

**Affiliations:** ^1^School of Pharmacy, College of Pharmacy, China Medical University, Taichung, 40402, Taiwan; ^2^Department of Fragrance and Cosmetic Science, College of Pharmacy, Kaohsiung Medical University, Kaohsiung 807, Taiwan; ^3^Department of Medicine, Mackay Medical College, Taipei 252, Taiwan; ^4^School of Pharmacy, College of Pharmacy, Kaohsiung Medical University, Kaohsiung 807, Taiwan; ^5^Department of Marine Biotechnology and Resources, College of Marine Sciences, National Sun Yat-sen University, Kaohsiung 80424, Taiwan

## Abstract

Nicotine is a potent chemical that excites the central nervous system and refreshes people. It is also physically addictive and causes dependence. To reduce the harm of tobacco products for smokers, a law was introduced that requires tobacco product containers to be marked with the amount of nicotine as well as tar. In this paper, an online stacking capillary electrophoresis (CE) method with cation-selective exhaustive injection sweeping-micellar electrokinetic chromatography (CSEI-sweeping-MEKC) is proposed for the optimized analysis of nicotine in tobacco. A higher conductivity buffer (160 mM phosphate buffer (pH 3)) zone was injected into the capillary, allowing for the analytes to be electrokinetically injected at a voltage of 15 kV for 15 min. Using 50 mM sodium dodecyl sulfate and 25% methanol in the sweeping buffer, nicotine was detected with high sensitivity. Thus, optimized conditions adapted from a chemometric approach provided a 6000-fold increase in the nicotine detection sensitivity using the CSEI-sweeping-MEKC method in comparison to normal CZE. The limits of detection were 0.5 nM for nicotine. The stacking method in combination with direct injection which matrix components would not interfere with assay performance was successfully applied to the detection of nicotine in tobacco samples.

## 1. Introduction

Nicotine, one of the predominant active components in tobacco, acts as both a relaxant and a stimulant, depending on the depth of puffs taken by smokers. It causes pharmacological responses in the whole body especially in the neural system [1]. Exposure to excessive amounts of nicotine may be pernicious to our health. Lately, there has been plenty of evidence from research showing that nicotine may increase the risk of suffering from Alzheimer's disease [2]. Some conspicuous evidence also indicates that neurological diseases, such as Parkinson's, and cardiovascular diseases may also be attributed to the effect of nicotine [3, 4]. Furthermore, nicotine is also the primary component in tobacco products that contributes to the addictive effect [5]. To reduce the damage of tobacco for smokers, a law was proposed that mandates that tobacco products should be marked with the amount of nicotine as well as tar and that the nicotine in each cigarette should not exceed more than 1.2 mg in Taiwan [6]. Thus, it is very essential to establish a method for determining the content of nicotine in tobacco.

Analysis of nicotine in cigarettes has been accomplished by several methods to date, including HPLC [7], radioimmunoassay [8], GC-MS [9, 10], and CE [11, 12]. Analytical methods using HPLC will be carried out in the presence of large amount of solvents, which may seem quite wasteful. The experiment conducted by radioimmunoassay is rapid and highly sensitive. However, the radioactive waste disposal may lead to potential health hazards and safety problems. Another important characteristic of nicotine is its volatility, which is suitable for the utilization of GC-MS. However, the relatively expensive apparatus of GC-MS does not offer a practical solution to many laboratories. Thus, we aimed to develop a fairly simple method by CE, not only because of its quick turn-around time but also because it is cost-effective in not using large amounts of solvents.

CE is well known for its high separation efficiency, great resolution, and low consumption of sample and reagents. However, the miniscule injection volume may lead to relatively lower sensitivity. Moreover, while using a UV detector, because the optical path length of the capillary is short, the absorbance is restricted. In the present study, we aim to develop an efficient online concentration method to determine nicotine content, particularly in tobacco, by utilizing CE coupled with cation-selective exhaustive injection sweeping-micellar electrokinetic chromatography (CSEI-sweeping-MEKC) to minimize the limitation in detection and manage the disadvantages mentioned above. CSEI-sweeping-MEKC was first established by Quirino and Terabe and introduced to identify samples containing 1-naphthylamine and to target cationic analytes [13]. The method was initially performed by electrokinetic injection of a cationic sample into a high conductive buffer, followed by the application of a surfactant to complete the sweeping procedure. The sample was therefore concentrated before being subject to the detection analysis. The results showed that the CSEI-sweeping-MEKC method provided a 5000-fold increase in UV detector response when compared to the traditional injection mode. Due to its high detection sensitivity, Wu et al. applied this technique to detect commonly abused drugs in human plasma, urine, and hair [14–17]. Xu and Fan used CSEI-sweeping-MEKC to determine cotinine in serum [18]. Overall, this technique is easily applicable in a lab setting without an additional apparatus and is also fairly cost-effective. Accordingly, we decided to adopt CSEI-sweeping-MEKC in our experiment to enhance the detection sensitivity.

Many factors need to be considered to acquire optimized condition, especially for complicated online preconcentration techniques. The most significant difference under optimized experimental conditions between traditional “one variable at a time” schemes and chemometric approach with experimental designs is that, in the former, researchers change one factor at a time to determine the optimal conditions, thereby requiring a large number of experiments that are independent, failing to consider interactions between factors. Chemometric approach provides a powerful statistical tool by reducing the number of required experiments, evaluating the interactions between factors and examining global experimental domains [19, 20]. Recently, chemometric approach with experimental designs was popularly applied in analytical chemistry techniques such as extraction procedures [21, 22] and separation experiments [23, 24]. The aim of this study was to investigate the impact of factors influencing the UV response, thus allowing the reduction of the number of factors to be optimized. The optimized methods can then be used to determine the content of nicotine in tobacco products.

## 2. Experimental

### 2.1. Materials

All chemicals were of analytical grade. Sodium dihydrogen phosphate (NaH_2_PO_4_), hydrogen chloride (HCl), sodium hydroxide (NaOH), and sodium dodecyl sulfate (SDS) were purchased from Merck (Darmstadt, Germany). Carbamazepine was used as an internal standard (IS) and was purchased from Sigma (St. Louis, USA). Methanol (MeOH) from Avantor (PA, USA) was used as the buffer additive. Deionized water (dd-H_2_O), prepared by a Milli-Q system (Millipore, Bedford, MA, USA), was used to prepare the buffers and related aqueous solutions. Three different nicotine-containing tobacco products (samples I, II, and III) were used in this study.

### 2.2. CE Conditions

A Beckman P/ACE MDQ system (Fullerton, CA, USA) with UV detector was used. This study was performed utilizing an uncoated fused-silica capillary (50 *μ*m, id) (Polymicro Technologies, Phoenix, AZ, USA) that had a length-to-detector of 40 cm and a total length of 50.2 cm. Before using a new capillary to run a separation, it was rinsed with a series of washes for 10 min each. The washes, in order, were the following: methanol, deionized water (dd-H_2_O), 1 M HCl, dd-H_2_O, 1 M NaOH, and dd-H_2_O at 30 psi. Between each experimental run, the column was rinsed with 1 M HCl for 5 min and dd-H_2_O for 5 min at 30 psi. The separation buffer consisted of a phosphate-HCl buffer (50 mM, pH 3.0) with 25% methanol. The high-conductivity buffer (HCB) is a phosphate-HCl buffer (160 mM, pH 3.0). The sweeping buffer is a phosphate-HCl buffer (50 mM, pH 3.0) containing sodium dodecyl sulfate (SDS, 50 mM) and 20% methanol. The HCB injection was achieved with a pressure of 1.5 psi over 99.9 s. The separation voltage was −25 kV, with the cathode at the detector end, and the temperature of the separation was maintained at 25°C. All operations, including the electropherogram acquisition, were computer controlled using the Beckman Coulter 32 Karat software system (Fullerton).

### 2.3. Sample Pretreatment

Tobacco products were ground into powder; 0.01 g of each tobacco powder was dissolved in 1 mL methanol, vortexed for 2 min, and followed by centrifugation (12000 rpm 10 min). The supernatant was filtered through a 0.45 *μ*m membrane filter and diluted 100-fold with dd-H_2_O. Stock solutions of IS were prepared at 1 mM in methanol. All stock solutions were suitably diluted with dd-H_2_O to obtain reference standards. The range over which the standard concentrations were quantitated for the calibration curves was 61.7–617 nM. The calibration curves were established with the corrected peak area ratio of each analyte to IS as ordinate (*Y*) and the concentration of each respective analyte in mM as abscissa (*X*). Three concentrations of each analyte were chosen for precision and accuracy analyses. The limits of detection (LOD) were determined with decreasing concentration of each analyte until the ratio of signal to noise equaled 3 (S/N = 3).

## 3. Results and Discussion

### 3.1. The Principle of CSEI-Sweeping-MEKC Mode

**Figure 1:**
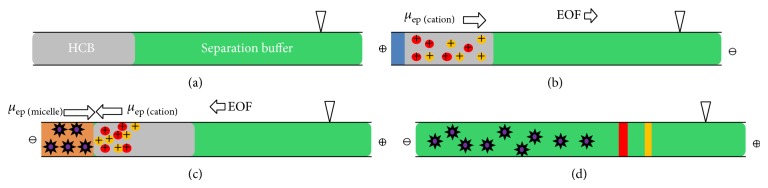
The mechanism of CSEI-sweeping-MEKC mode (adapted from [13]). (a) The capillary was rinsed with a nonmicellar, low-pH separation buffer. HCB zone was then introduced hydrodynamically. (b) Cationic samples prepared in low conductive matrix were injected into capillary by positive voltage and concentrated in the HCB zone. (c) The concentrated cationic samples in HCB zone were then swept into a narrow zone by anionic micelles from the inlet sweeping buffer. (d) MEKC separation.

CSEI-sweeping-MEKC mode combines two preconcentration techniques in capillary electrophoresis. The principle of CSEI-sweeping-MEKC mode is shown in Figure 1. The capillary was first filled with a nonmicellar, low-pH separation buffer. A zone of high-conductivity buffer (HCB) was then injected hydrodynamically (Figure 1(a)). The cationic samples prepared in a low conductive matrix were then electrokinetically injected by positive voltage for a specific time period (e.g., 15 min). The cationic samples were concentrated in the HCB zone due to the large difference in electric field strength between the sample matrix and HCB (Figure 1(b)). After sample injection, a negative voltage was applied and the capillary was placed with both ends in the sweeping buffer, which contained anionic micelles. The concentrated cationic samples in the HCB zone were swept into a narrow zone by anionic micelles from the inlet sweeping buffer (Figure 1(c)). Finally, the separation was accomplished by MEKC (Figure 1(d)) [13].

### 3.2. Preliminary Experiments

There are many factors that should be discussed in the “one variable at a time” traditional optimized procedure of CSEI-sweeping-MEKC mode. However, some factors may only have a small effect. Hence, some parameters were evaluated for their influence on detection sensitivity and resolution in preliminary experiments to make sure that they are suitable for inclusion in the next step, experimental design. First, phosphate was chosen as both the separation and sweeping buffer because of its low-pH value that can reduce the EOF. If the EOF is too large, the samples will be easily pushed back into the inlet buffer vial or the migration time will be too long when applying a negative voltage. When the pH value of phosphate buffer was set as 2, 3, and 4, the peak of nicotine was clearly found in the electropherograms, and no significant difference in sensitivity was observed under these conditions. Therefore, the pH value of phosphate was set at 3 in the preliminary experiments. When the buffer concentrations were lower than 100 mM, sufficient resolution and sensitivity were observed in the electropherograms. Meanwhile, the current and Joule heat need to considered. Finally, 50 mM was chosen as the buffer concentration. Other factors such as the methanol proportion in the separation buffer, SDS concentration in the sweeping buffer, and HCB phosphate concentration could play an important role in the CSEI-sweeping-MEKC mode. Hence, they were included in the chemometric approach for discussion.

### 3.3. Experimental Design and Data Analysis

A central composite design, which is composed of a two-level full factorial design, star design, and two or more center points, was the final step for acquiring the optimized separation condition. Two or more center points were used to evaluate the reproducibility of analytical performance. Central composite design is useful in investigating the linear, quadratic, and cross effects of these three factors [25]. The settings of the factors in the design are provided in Table 1. Each is varied at five levels (−*α*, −1, 0, + 1, + *α*), and two center points for replication are included in 16 experimental runs. Alfa level is equal to (2^*f*^)^1/4^, where *f* is the number of factors. When all three factors, including methanol proportion, SDS concentration, and HCB phosphate concentration, were examined, *α* was equal to 1.68 [26]. The conditions of 16 experimental runs are shown in Table 2. In order to evaluate the sensitivity enhancement of CSEI-sweeping-MEKC, peak height was chosen to calculate the response of CCD. Meanwhile, the highest peak in 16 experimental runs was set at 100. The data involved in these calculations are shown in Table 3. To evaluate the relationship between the three parameters and the peak height, a regression equation was established as follows:

(1) y=A0+A1×X1+A2×X2+A3×X3+A4×X1×X2+A5×X2×X3+A6×X1×X3+A7×X1×X1+A8×X2×X2+A9×X3×X3,

where *Y* is the peak height; *X*_1_ is the methanol proportion; *X*_2_ is the SDS concentration; *X*_3_ is the HCB phosphate concentration; *A*_0_ is the regression coefficient; *A*_1_, *A*_2_, and *A*_3_ are linear coefficients; *A*_4_, *A*_5_, and *A*_6_ are cross-effect coefficients; and *A*_7_, *A*_8_, and *A*_9_ are quadratic coefficients. The experimental results were fitted to the proposed regression model using the JMP software, and the equation model for peak height was as follows:

(2) Peak  height=48.61+20.3X1+1.21X2+11.39X3−5.52X1×X2−4.82X1×X3+1.22X2×X3+8.56X1×X1+5.78X2×X2−5.79X3×X3.

**Figure 2:**
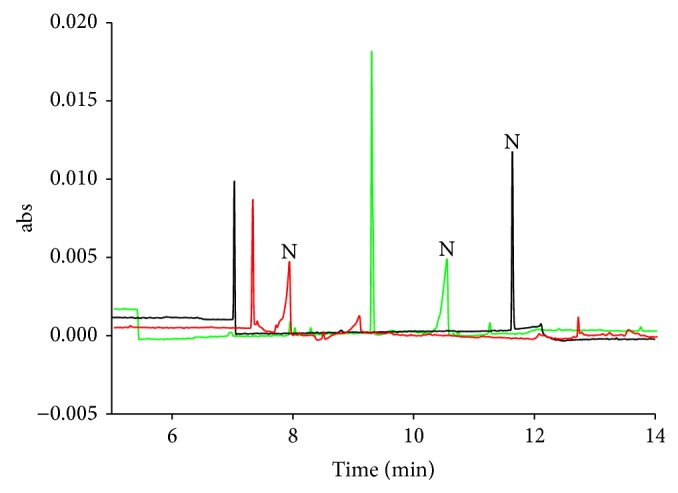
Electropherograms of runs 10 (blank line), 13 (red line), and 15 (green line) in CCD. CE conditions: separation buffer: 50 mM phosphate buffer (pH 3) containing methanol; sweeping buffer: 50 mM phosphate buffer (pH 3) containing 20% methanol and SDS; HCB: phosphate buffer (pH 3); applied voltage, −20 kV. Sample injection: 15 kV, 15 min. Nicotine concentration: 617 nM. Other conditions are shown in Table 2.

According to the ANOVA results (Table 3), the coefficient-of-determination was 0.95, indicating that the equations are reliable. The methanol proportion (*X*_1_), SDS concentration (*X*_2_), and HCB phosphate concentration (*X*_3_) have positive coefficients. The *P* values of these *X*_1_, *X*_2_ were less than 0.05. This indicates that the methanol proportion and HCB phosphate concentration influence the detection efficiency and contribute significantly to the model. In addition, no interaction between *X*_1_, *X*_2_, and *X*_3_ was found, and all *P* values were above 0.05. The coefficient for *X*_1_ × *X*_1_ was among the highest calculated. This indicates that the methanol proportion had a quadratic effect on the response. From Figure 2, it is clear that the smallest peak width and highest peak height were observed in run 10.

**Figure 3:**
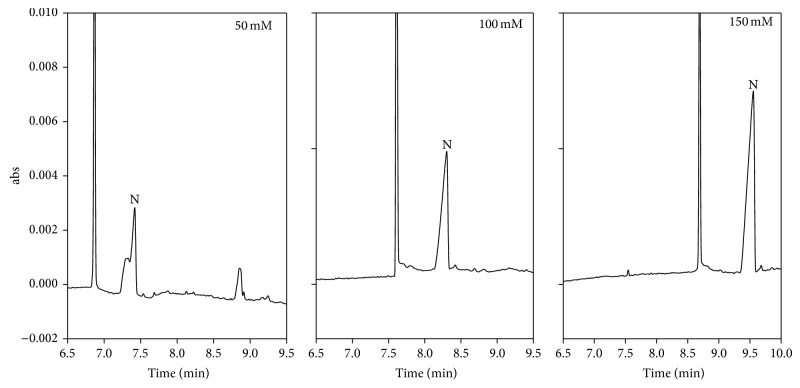
Effect of HCB concentration by CSEI-sweeping-MEKC. Separation buffer: 50 mM phosphate buffer (pH 3) containing 20% methanol; sweeping buffer: 50 mM phosphate buffer (pH 3) containing 20% methanol and 50 mM SDS; HCB: phosphate buffer (pH 3); applied voltage, −20 kV. Sample injection: 15 kV, 15 min. Nicotine concentration: 617 nM.

The main reason for adding methanol into the separation buffer is to decrease the polarity of buffer, which affects the partition equilibrium of nicotine among the micelles and aqueous buffer. When adding 0–5% methanol, nicotine was not observed. The blank peak and nicotine have the same electrophoretic mobility. When the methanol proportion was increased to 25%, higher resolution and detection sensitivity were acquired. The different SDS levels in phosphate buffer (50 mM, pH 3) were evaluated for their sweeping efficiency. Anionic micelles, SDS, were introduced into the capillary when applying a negative voltage. The electrophoretic mobility of SDS was faster than the nicotine ion, so that the SDS can sweep the stacked nicotine ion to increase detection sensitivity. However, different SDS levels have no significant effect. The HCB zone (1.5 psi for 99.9 s) was introduced into the capillary after the separation buffer. After theexhaustive electrokinetic sample injection, nicotine ion slows down and stacks at the front boundary of HCB because of the large difference in the electric field strength between the sample matrix and HCB. The higher HCB concentration can reduce the electrophoretic mobility of nicotine and stack the cations. When HCB concentration was set at 50 mM, the worst stacking efficiency was observed. When the HCB concentration was increased to 150 mM, higher resolution and detection sensitivity were acquired. The results are shown in Figure 3.

**Figure 4:**
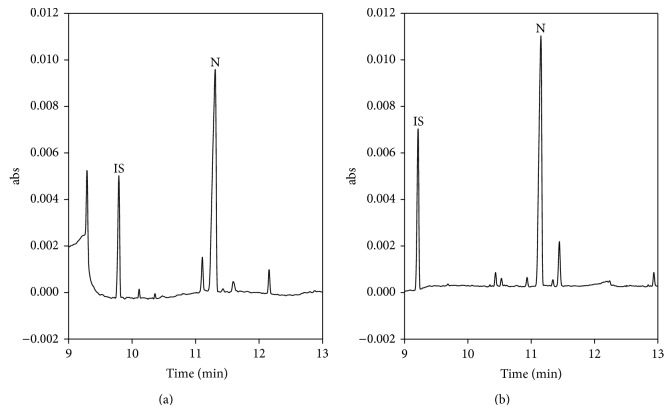
Electropherograms for determining nicotine (standard solution) under optimized conditions (a) and tobacco sample I (b). CE conditions: separation buffer: 50 mM phosphate buffer (pH 3) containing 25% methanol; sweeping buffer: 50 mM phosphate buffer (pH 3) containing 20% methanol and 50 mM SDS; HCB: 160 mM phosphate buffer (pH 3); applied voltage, −25 kV. Sample injection: 15 kV, 15 min.

The CCD results predicted a 25% methanol proportion, 50 mM SDS, and 160 mM HCB phosphate concentration as optimal conditions by JMP software. The electropherogram of the optimized CE condition was shown in Figure 4(a). Compared with the detection efficiency acquired by the “one variable at a time” method under optimized conditions (20% methanol proportion, 50 mM SDS, and 100 mM HCB phosphate concentration), detection sensitivity was improved significantly in our experiments. The ratio of peak height between CCD and “one variable at a time” was 1.45. The results clearly showed that the CCD can predict better CE conditions to improve detection sensitivity.

### 3.4. LOD and Linearities

To evaluate the quantitative applicability of the established CE method in tobacco samples, five different concentrations of nicotine were analyzed using carbamazepine (500 ng/mL) acting as an IS to calculate the peak area ratios by dividing the corrected peak areas of each analyte. The corrected peak area that was used with the standard was calculated as follows: peak area of an analyte/migration time of the analyte divided by the peak area of IS/migration time of IS. Calibration curves were obtained from linear peak area ratios with the *X* axis as concentration and the *Y* axis as peak area ratios. The regression equations was *Y* = (0.0324 ± 0.0014)*X* + (−0.0354 ± 0.0022) with high linearities, and the correlation coefficient (*r*) of each curve was greater than 0.998 (*n* = 3). The recoveries were calculated on the analyses of nicotine (at levels of 20, 60, and 190 ng/mL). The results indicated that the recovery values were 99.3–110.6%, with the LOD = 0.5 ng/mL (S/N = 3). Thus, a 6000-fold enhancement was observed in detection sensitivity when using the CSEI-sweeping-MEKC method compared to the normal CZE (LOD: 3 *μ*g/mL). All data hereby indicate that the developed method can estimate concentration values using this online preconcentration method.

### 3.5. Application

Because nicotine can easily cause damage in and is highly additive to the human body, it was mandated that the nicotine content in tobacco products should be regulated. Combined with a 1000-fold dilution of sample and direct injection, which can decrease the interference of matrix effect, the CSEI-sweeping-MEKC method was used to determine the nicotine levels in tobacco. The nicotine content of three tobacco samples (samples I, II, and III) was determined to be 0.3 mg, 0.4 mg, and 0.8 mg per tobacco sample, respectively, by CSEI-sweeping-MEKC method. The electropherograms obtained from the analysis of sample I is shown in Figure 4(b). The compositions of all the samples were further confirmed by GC-MS. The quantitation of nicotine in samples I, II, and III were 0.28 ± 0.02 mg, 0.42 ± 0.03 mg, and 0.73 ± 0.06 mg per tobacco sample, respectively. Consequently, the CSEI-sweeping-MEKC method can be successfully used for the determination of nicotine levels in tobacco.

## 4. Conclusions

The online preconcentration method, CSEI-sweeping-MEKC, and central composite design were successfully applied to determine nicotine levels in tobacco with optimized detection efficiency. Some factors, including methanol proportion and HCB phosphate concentration, significantly affected the detection sensitivity, which was verified by ANOVA. Compared to the CZE mode, CSEI-sweeping-MEKC provides a signal enhancement of 6000-fold. Three tobacco samples with different nicotine concentrations were successfully analyzed with excellent sensitivity by the online preconcentration “CSEI-sweeping-MEKC” technology. In the future, we would like to apply this method to determine nicotine and its metabolites in plasma.

## Figures and Tables

**Table 1:** Values of CCD variables.

	−*α*	−1	0	+1	+*α*
Methanol proportion (%)	4.88	10	17.5	25	30.11
SDS concentration (mM)	24.43	50	87.5	125	150.56
HCB phosphate concentration (mM)	32.38	75	137.5	200	242.61

**Table 2:** Experimental design for CCD.

Run	Pattern	Methanol proportion	SDS concentration	HCB phosphate concentration	Peak high (%)
1	− − −	10	50	75	20.38
2	− − +	10	50	200	40.73
3	− + −	10	125	75	15.73
4	− + +	10	125	200	63.53
5	+ − −	25	50	75	75.41
6	+ − +	25	50	200	99.02
7	+ + −	25	125	75	71.26
8	+ + +	25	125	200	77.17
9	a 0 0	4.88	87.50	137.50	43.64
10	A 0 0	30.11	87.50	137.50	100
11	0 a 0	17.50	24.43	137.50	56.68
12	0 A 0	17.50	150.56	137.50	71.24
13	0 0 a	17.50	87.50	32.38	14.00
14	0 0 A	17.50	87.50	242.61	48.41
15	0 0 0	17.50	87.50	137.50	52.04
16	0 0 0	17.50	87.50	137.50	48.55

**Table 3:** ANOVA parameters for peak height.

Source	Sum of squares	df	Mean squares	*F* value	*P* value
Model	9842.34	9	1093.59	12.75	0.0029^*∗*^
*X*_1_	5629.57	1	5629.57	65.66	0.0002^*∗*^
*X*_2_	20.26	1	20.26	0.23	0.6441
*X*_3_	1771.48	1	1771.48	20.66	0.0039^*∗*^
*X*_1_ *X*_2_	0.034	1	243.65	243.65	0.1428
*X*_1_ *X*_3_	0.025	1	186.53	2.17	0.1906
*X*_2_ *X*_3_	1.006	1	11.88	0.13	0.7225
*X*_1_^2^	0.806	1	679.73	7.92	0.0305^*∗*^
*X*_2_^2^	0.110	1	310.23	3.61	0.1058
*X*_3_^2^	0.484	1	310.97	3.62	0.1055^*∗*^
	*R* ^2^		0.950		

*X*_1_: methanol proportion; *X*_2_: SDS concentration; *X*_3_: HCB phosphate concentration.

*R* ^2^: coefficients of determination that totally explain the variance in the data.

^*∗*^ *P* < 0.05.
